# Comparative clinical features and short-term outcomes of gastric and small intestinal gastrointestinal stromal tumours: a retrospective study

**DOI:** 10.1038/s41598-019-46520-1

**Published:** 2019-07-11

**Authors:** Zhengyang Yang, Feng Wang, Song Liu, Wenxian Guan

**Affiliations:** Department of General Surgery, Drum Tower Hospital, Medical School of Nanjing University, 321 Zhongshan RD, Nanjing, 210008 China

**Keywords:** Gastrointestinal cancer, Gastric cancer, Intestinal diseases

## Abstract

Gastrointestinal stromal tumours (GISTs) are the most common mesenchymal tumours of the gastrointestinal tract. Recent research has shown that small intestinal GISTs exhibit more aggressive features than gastric GISTs. To compare the clinical features of gastric and small intestinal GISTs for the further prediction of different prognoses, we conducted a retrospective study. 43 patients in the small intestine group and 97 in the gastric group were collected between January 2016 and December 2017. Data on demographics, preoperative lab results, clinicopathological results and surgical management were compared between groups. Significant elements were subsequently included in logistic regression analysis for further identification. The Kaplan-Meier method and log-rank test were used to calculate the relapse-free survival (RFS) rate and cumulative survival rate. Univariable analysis demonstrated that underlying disease, gastrointestinal (GI) bleeding, lymphocyte count, haemoglobin (Hb), albumin (ALB), platelet-to-lymphocyte ratio (PLR), thrombin time (TT), National Institutes of Health (NIH) category, Dog1, surgical procedure types and postoperative hospitalization were different between the two groups. Among these factors, logistic regression analysis identified that patients in small intestinal group exhibited significantly higher GI bleeding rate (p = 0.022), NIH category (p = 0.031), longer postoperative hospitalization time (p = 0.001) with lower TT value (p = 0.030) than those in gastric group. The log-rank test indicated that the location of the GIST (p = 0.022), GIST with GI bleeding (p = 0.027) and NIH category (p = 0.031) were independent prognostic predictors for poor outcome regarding RFS. Regarding cumulative survival, only the location of the GIST (p = 0.027) was an independent prognostic predictor for poor outcome. Thus, we concluded that small intestine GISTs were associated with lower TT, recurrent GI bleeding, advanced NIH category and extended postoperative hospitalization. Nevertheless, future multicentre prospective study are expected to validate our results.

## Introduction

GISTs are the most common mesenchymal tumours in the gastrointestinal tract^1,2^. Oncogenic mutations in KIT (CD117) or platelet‐derived growth factor receptor alpha (PDGFRα) genes may cause GISTs^3,4^. GISTs can occur in any part of the gastrointestinal tract, of which the most common site is the stomach (50–60%), followed by the small intestine (20–30%), duodenum (4–5%), rectum (4%), colon and appendix (1–2%)^5^. GISTs rarely occur outside of the gastrointestinal tract but occasionally form in locations such as the omentum, mesentery or retroperitoneum^6^.

It is a common dogma that small intestinal GISTs have a worse prognosis than gastric GISTs because of the higher risk of metastases and tumour-related death^7^. The widely used Armed Forces Institute of Pathology (AFIP) classification refers to GIST location as a third risk factor^8^. A recent study comprising 5,607 patients with gastric and small intestinal GISTs demonstrated that small intestinal primaries exhibited aggressive features such as high pathological grade and large size^9^. On the other hand, the MSKCC (Memorial Sloan Kettering Cancer Center) found that patients with small intestinal GISTs were statistically similar to patients with gastric GISTs in terms of cancer-specific survival (CSS) and overall survival (OS)^10^. These findings may have tremendous implications for evaluating the different risks between gastric and small intestinal GISTs.

Due to the increasing incidence of GISTs, we aimed to compare clinical features between gastric and small intestine GISTs and subsequently identify significant factors associated with clinical prognosis.

## Materials and Methods

### Patient selection

Between January 2016 and December 2017, all patients with gastric and small intestine GISTs that registered in the gastrointestinal surgery unit of our hospital were collected for qualification screening. The inclusion criteria of this study were as follows: (1) absence of recurrent or metastatic GISTs; (2) absence of other malignant diseases; (3) received R0 resection by open or laparoscopic surgery; (4) absence of preoperative treatment including imatinib, chemotherapy and radiotherapy.

### Data collection

Demographics, preoperative lab results, clinicopathological results and surgical management data were retrieved from the Electronic Medical Record System. Demographics included sex, age, hypertension, diabetes, other underlying diseases, past abdominal surgical history, tobacco usage, and alcohol usage, with gastrointestinal (GI) bleeding as the chief complaint. The preoperative lab results included white blood cell (WBC), neutrophil, lymphocyte, and monocyte counts, haemoglobin (Hb), platelet (PLT), albumin (ALB), and C-reactive protein (CRP) levels, neutrophil-to-lymphocyte ratio (NLR), platelet-to-lymphocyte ratio (PLR), faecal occult blood test, alpha-fetoprotein (AFP), carcinoembryonic antigen (CEA), CA125, CA724, CA242, prothrombin time (PT), activated partial prothrombin time (APPT) and thrombin time (TT). All these results were derived from the final blood test results before surgery. The NLR value was calculated as the neutrophil count (number of neutrophils/mL) divided by the lymphocyte count (number of lymphocytes/mL). The PLR value was calculated in the same manner as the NLR. The clinicopathological data included lymph node involvement, tumour size, mitotic index, the National Institutes of Health (NIH) category, CD117, CD34, Ki67, Dog1 and SDHB. The NIH category was collected according to the NIH consensus classification system, which is now widely used as a risk stratification scheme for GISTs^11^. All antibody including AFP, CEA, CA125, CA199, CA724, CA242, CD117, CD34, Ki67, Dog1 and SDHB were purchased from Nanjing KeyGEN Biotechnology (Nanjing, China). Surgical management involved surgical procedure, operation time, intraoperative haemorrhage, postoperative hospitalization time and postoperative complications. All included patients were divided into gastric or small intestine groups according to the site of the malignancy. Patients with small intestinal GISTs included duodenal, jejunal and ileal GISTs.

### Statistical analysis

All analyses were 2-tailed. The confidence interval was 5–95%, and p-values < 0.05 were considered to be statistically significant. Univariate analysis was performed using different methods according to different data types. Categorical variables were analyzed using chi-square test with Fisher’s exact test and were presented as frequencies (percentages). Continuous variables were analyzed using an unpaired t-test with Welch’s correction and were presented as the mean ± SD (standard deviation). Further multivariate analysis was performed using logistic regression analysis. Significant differences between the 2 groups in univariate analysis were assessed into the multivariate analysis to identify the independent influencing factors. The Kaplan-Meier method and log-rank test were used to calculate the relapse-free survival (RFS) rate and cumulative survival rate. All the above analyses were performed using SPSS software (version 24.0; IBM Inc., Chicago, IL).

### Follow-up

Follow-up data collection was performed by professional researchers through telephone and regular outpatient visits. The follow-up period was calculated from the date of surgery to the date of recurrence, metastasis or the latest follow-up date. Eventually, 24 patients were lost and the remaining 116 patients were finally enrolled.

### Ethics

This study was approved by the Ethics Committees of Nanjing Drum Tower Hospital. As a retrospective study, informed consent is not required from participants.

## Results

Between January 2016 and December 2017, 140 patients with GISTs who received surgery were enrolled in this study, including 43 patients (30.71%) in the small intestine group and 97 (69.29%) in the gastric group.

### Demographic data

As shown in Table 1, there were no significant difference in gender, age, hypertension, diabetes, past abdominal surgical history, tobacco usage and alcohol usage. Patients with other underlying diseases including 8 patients with lacunar cerebral infarction, 4 patients with coronary disease, 4 patients with chronic bronchitis, 2 patients with atherosclerosis, 1 patient with parkinsonism and 1 patient with renal inadequacy. Meanwhile, patients in small intestinal group exhibited a higher incidence rate of other underlying diseases than those in gastric group (p = 0.043). GI bleeding was more common in small intestinal GIST patients than in the gastric GIST patients (p = 0.001).

**Table 1:** Comparison of small intestinal GIST and gastric GIST patients in patient demographics, preoperative lab test, clinopathological results and Surgical management data.

	total (n = 140)	small intestinal GIST (n = 43)	gastric GIST (n = 97)	P-value
Male (n, %)	72(51.4%)	28(65.10%)	44(45.40%)	0.051
Age (Median ± SD)	60.26 ± 11.01	58.33 ± 10.02	60.63 ± 11.46	0.645
Hypertension (n, %)	51(36.4%)	12(27.9%)	39(40.2%)	0.163
Diabetes (n, %)	16(11.4%)	2(4.7%)	14(14.4%)	0.093
Other underlying disease (n, %)	20(14.3%)	10(23.3%)	10(10.3%)	0.043*
Past abdominal surgical history (n, %)	44(31.4%)	15(34.9%)	29(29.9%)	0.558
Tobacco usage (n%)	19(13.6%)	8(18.6%)	11(11.3%)	0.247
Alcohol usage (n%)	15(10.7%)	7(16.3%)	8(8.2%)	0.156
Gastrointestinal bleeding as chief complaint (n%)	47(33.6%)	23(53.5%)	24(24.7%)	0.001*
WBCs (Median ± SD) 10^9/L	5.40 ± 3.29	5.10 ± 2.23	5.62 ± 3.68	0.333
Neutrophils (Median ± SD) 10^9/L	3.19 ± 3.33	3.11 ± 2.26	3.31 ± 3.74	0.703
Lymphocytes (Median ± SD) 10^9/L	1.48 ± 0.53	1.24 ± 0.42	1.53 ± 0.54	<0.001*
Monocytes (Median ± SD) 10^9/L	0.35 ± 0.17	0.33 ± 0.17	0.36 ± 0.17	0.27
Hb (Median ± SD) g/L	117.07 ± 25.93	105.60 ± 25.89	124.25 ± 25.40	0.028*
PLT (Median ± SD) 10^9/L	202.12 ± 72.19	205.00 ± 73.93	200.67 ± 71.28	0.267
ALB (Median ± SD) g/L	39.33 ± 3.46	38.90 ± 4.08	40.05 ± 3.05	0.040*
CRP (Median ± SD) mg/L	3.14 ± 11.86	3.10 ± 14.72	3.15 ± 10.20	0.143
NLR (Median ± SD)	3.22 ± 6.01	2.40 ± 3.15	2.04 ± 6.97	0.962
PLR (Median ± SD)	161.41 ± 95.49	174.60 ± 90.97	123.27 ± 95.13	0.018*
Faecal occult blood test				0.163
−(n%)	95(67.9%)	27(62.8%)	68(70.1%)	—
+ (n%)	23(16.4%)	10(23.3%)	13(13.4%)	—
Unknown(n%)	22(15.7%)	6(14.0%)	16(16.5%)	—
AFP (Median ± SD) ng/ml	2.06 ± 2.29	2.25 ± 3.24	1.98 ± 1.70	0.23
CEA (Median ± SD) ng/ml	1.36 ± 6.43	1.02 ± 9.37	1.43 ± 4.59	0.42
CA125 (Median ± SD) U/ml	8.62 ± 42.98	8.20 ± 21.94	9.47 ± 49.75	0.706
CA199 (Median ± SD) U/ml	8.17 ± 17.81	7.32 ± 26.33	8.97 ± 12.31	0.536
CA724 (Median ± SD) U/ml	1.05 ± 3.43	1.02 ± 1.67	1.10 ± 3.96	0.281
CA242 (Median ± SD) U/ml	3.47 ± 3.16	2.83 ± 3.21	3.79 ± 3.14	0.329
PT (Median ± SD) s	11.91 ± 1.66	11.96 ± 0.88	11.86 ± 1.91	0.453
APPT (Median ± SD) s	28.29 ± 5.35	27.93 ± 3.65	28.75 ± 5.93	0.115
TT (Median ± SD) s	18.60 ± 2.08	17.88 ± 1.68	18.80 ± 2.21	0.041*
Lymph node involvement				0.223
No (n%)	137(97.9%)	41(95.3%)	96(99.0%)	—
Yes (n%)	3(2.1%)	2(4.7%)	1(1.0%)	—
Tumor size	5.53 ± 3.15	5.80 ± 2.97	5.41 ± 3.23	0.752
Tumor size classification				0.317
<5 cm (n%)	74(52.9%)	20(46.5%)	54(55.7%)	—
≥5 cm (n%)	66(47.1%)	23(53.5%)	43(44.3%)	—
Mitotic index				0.471
<5/50 HPF (n%)	75(53.6%)	25(58.1%)	50(51.5%)	—
≥5/50 HPF (n%)	65(46.4%)	18(41.9%)	47(48.5%)	—
NIH category				<0.001*
Very low (n%)	8(5.7%)	1(2.3%)	7(7.2%)	—
Low (n%)	58(41.4%)	15(34.9%)	43(44.3%)	—
Intermediate (n%)	25(17.9%)	1(2.3%)	24(24.7%)	—
High (n%)	49(35.0%)	26(60.5%)	23(23.7%)	—
CD117				0.22
−(n%)	12(8.6%)	1(2.3%)	11(11.3%)	—
+ (n%)	24(17.1%)	7(16.3%)	17(17.5%)	—
++ (n%)	43(30.7%)	12(27.9%)	31(32.0%)	—
+++ (n%)	61(43.6%)	23(53.5%)	38(39.2%)	—
CD34				0.459
−(n%)	20(14.3%)	6(14.0%)	14(14.4%)	—
+ (n%)	24(17.1%)	9(20.9%)	15(15.5%)	—
++ (n%)	31(22.1%)	12(27.9%)	19(19.6%)	—
+++ (n%)	65(46.4%)	16(37.2%)	49(50.5%)	—
Ki67				0.662
<5%+ (n%)	69(49.3%)	20(46.5%)	49(50.5%)	—
≥5%+ (n%)	71(50.7%)	23(53.5%)	48(49.5%)	—
Dog1				0.008*
−(n%)	3(2.1%)	1(2.3%)	2(2.1%)	—
+ (n%)	19(13.6%)	2(4.7%)	17(17.5%)	—
++ (n%)	31(22.1%)	5(11.6%)	26(26.8%)	—
+++ (n%)	87(62.1%)	35(81.4%)	52(53.6%)	—
SDHB				0.215
−(n%)	8(5.7%)	4(9.3%)	4(4.1%)	—
+ (n%)	69(49.3%)	16(37.2%)	53(54.6%)	—
++ (n%)	38(27.1%)	13(30.2%)	25(25.8%)	—
+++ (n%)	25(17.9%)	10(23.3%)	15(15.5%)	—
Surgical procedure				<0.001*
Laparoscopic operation (n, %)	53(37.9%)	7(16.3%)	46(47.4%)	—
Laparotomy (n, %)	87(62.1%)	36(83.7%)	51(52.6%)	—
Operation time (Median ± SD)	139.57 ± 71.93	140 ± 85.24	139.17 ± 64.59	0.108
Intraoperative haemorrhage (Median ± SD)	78.50 ± 502.34	84.62 ± 874.39	71.60 ± 150.34	0.23
Postoperative hospitalization time (Median ± SD)	10.71 ± 6.80	10.83 ± 9.56	8.45 ± 4.38	0.002*
Postoperative complications (n, %)	11(7.9%)	5(11.6%)	6(6.2%)	0.313

Standard deviation (SD), white blood cell (WBC), haemoglobin (Hb), platelet (PLT), albumin (ALB), C reactive protein (CRP), neutrophil-to-lymphocyte ratio NLR), platelet-to-lymphocyte ratio (PLR), alpha fetoprotein (AFP), carcinoembryonic antigen (CEA), prothrombin time (PT), activated partial prothrombin time (APPT), thrombin time (TT), national institutes of health (NIH).

### Preoperative lab test results

The routine preoperative blood test showed lower lymphocyte counts (p < 0.001), Hb (p = 0.028) and ALB (p = 0.040) in the small intestine group. The PLR in the small intestine group was higher than in the gastric group (p = 0.018). The coagulation function results showed longer TT in the gastric group (p = 0.041). No significant differences were found in other data (Table 1).

### Clinicopathological results

Regarding the clinicopathology, our analysis showed that the small intestine group had a higher risk classification according to the NIH consensus classification system (p < 0.001). Furthermore, the small intestine group showed a stronger positive Dog1 level than the gastric group (p = 0.008). No significant differences were found in other data (Table 1).

### Surgical management data

According to the surgical management data, the surgeon preferred laparoscopic methods when operating on the small intestinal GIST patients (p < 0.001). The small intestine group had a longer postoperative hospitalization time than the gastric group (p = 0.002). No significant differences were found in other data (Table 1).

### Logistic regression analysis

To remove the interference from intrinsic relationship and interaction between variables, binary logistic regression analysis was subsequently performed to control potential confounding variables thus identifying significant elements between two groups. Elements that were significant in previous univariate analyses, including other underlying diseases, GI bleeding, lymphocyte counts, Hb, ALB, PLR, TT, NIH category, Dog1, surgical procedure and postoperative hospitalization time, were included in the logistic regression model. The results demonstrated that GI bleeding (p = 0.022, OR = 0.168, 95% CI: 0.036–0.777), TT (p = 0.030, OR = 1.315, 95% CI: 1.027–1.648), NIH category (p = 0.031) and postoperative hospitalization (p = 0.001, OR = 0.831, 95% CI: 0.749–0.922) were significantly different between the small intestine and gastric groups (Table 2).

**Table 2:** Multivariate analysis for patients with gastric GIST and patients with small intestinal GIST.

	OR	95% CI	p-value
Other underlying disease	0.597	0.145–2.466	0.476
Gastrointestinal bleeding	0.168	0.036–0.777	0.022*
Lymphocyte	1.41	0.327–6.079	0.645
Hb	0.984	0.958–1.012	0.263
ALB	0.954	0.809–1.124	0.571
PLR	1.003	0.995–1.011	0.466
TT	1.315	1.027–1.684	0.030*
NIH category			0.031*
Very low	ref		
Low	0.049	0.000–10.835	0.274
Intermediate	0.147	0.000–43.431	0.509
High	0.013	0.000–2.970	0.117
Dog1			0.132
−	ref		
+	4.417	0.168–116.081	0.373
++	2.421	0.131–44.785	0.552
+++	0.566	0.040–8.014	0.674
Surgical procedure	0.734	0.228–2.368	0.605
Postoperative hospitalization time	0.831	0.749–0.922	0.001*

Haemoglobin (Hb), albumin (ALB), platelet-to-lymphocyte ratio (PLR), thrombin time (TT).

### Tumour relapse and survival analysis

Among the 140 GIST patients, 24 patients were lost to follow-up, and 116 patients were finally followed up and evaluated in the relapse and survival analysis. During the follow-up period, 8 patients experienced tumour recurrence, while 6 in small intestinal group and 2 in gastric group. No small intestinal GIST patient and 1 gastric GIST patient experienced tumour metastasis. Unfortunately, 5 patients died ultimately all because of the tumour recurrence with 4 in small intestinal group and 1 in gastric group. The time to recurrence ranged from 4 to 24 months (median, 15 months), and the time to death ranged from 9 to 29 months (median, 20 months). According to our follow-up data, the RFS rate of the 116 GIST patients was 93.1%, and the cumulative survival rate was 95.7%.

**Figure 1:**
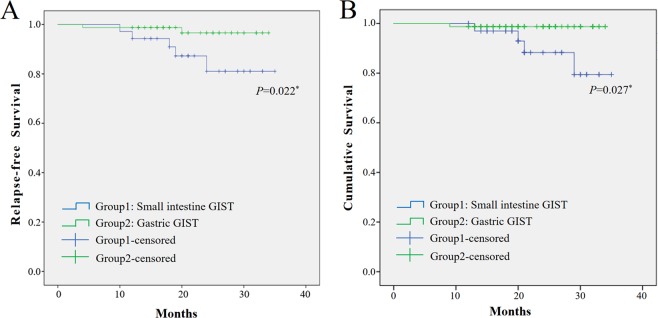
Kaplan-Meier curve analysis demonstrated worse relapse-free survival and cumulative survival for patients presenting with small intestine GIST.

**Figure 2:**
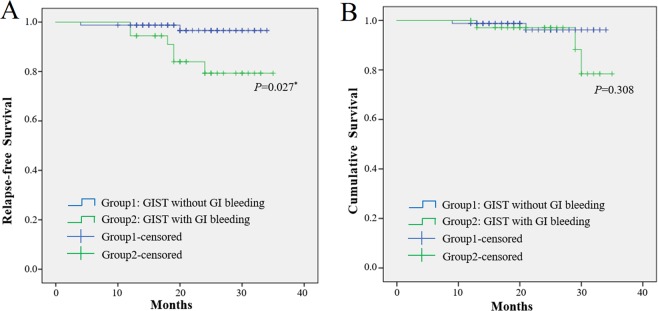
Kaplan-Meier curve analysis demonstrated worse relapse-free survival for GIST patients presenting with GI bleeding.

**Figure 3:**
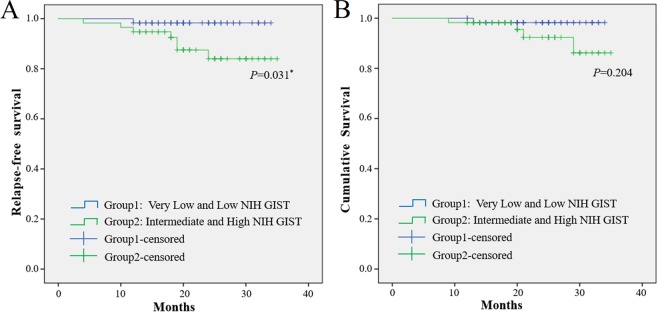
Kaplan-Meier curve analysis demonstrated worse relapse-free survival for GIST patients presenting with very low and low NIH GIST.

The Kaplan-Meier method and log-rank test were used to confirm the prognostic factors and showed that the gastric GIST patients had a greater RFS rate and cumulative survival rate (p = 0.022) than patients with small intestinal GISTs (p = 0.027, Fig. 1). Furthermore, according to the above logistic regression analysis, we assessed the associations of GI bleeding and NIH category with risk of relapse and survival. As shown in Fig. 2, GIST patients with GI bleeding were at increased risk of tumour relapse compared with those without GI bleeding (p = 0.027), while there were no significant differences in the cumulative survival rate (p = 0.308). In addition, the risk for relapse in the patients in the intermediate and high NIH groups was significantly increased compared with that in the very low and low NIH groups (p = 0.031). However, no significant differences were observed in the cumulative survival rate (p = 0.204, Fig. 3).

## Discussion

Herein, we summarize the main findings of our study by comparing 43 small intestinal GIST and 97 gastric GIST patients from January 2016 to December 2017. We discovered that patients with small intestinal GISTs might be more likely to have accompanying underlying diseases and GI bleeding. These patients may also exhibit lower preoperative lymphocyte counts, Hb, ALB, and TT levels and a higher PLR. Additional factor differences between the two groups included NIH category, Dog1 level, surgical procedure and postoperative hospitalization time. Logistic regression analysis identified that GI bleeding, TT, NIH category and postoperative hospitalization were significantly different between the small intestine and gastric groups. Furthermore, survival analysis demonstrated that the location of the GIST, GI bleeding and NIH category were independent prognostic predictors for poor outcome regarding RFS, while only the location of the GIST was an independent prognostic predictor for cumulative survival rate.

GI bleeding is one of the most common clinical manifestations of GIST^12^. Studies have indicated that in digestive tract GISTs, GI bleeding is the first symptom in 17% to 53% of cases, and manifests as haematemesis, black stool, faecal occult blood and with or without anaemia^13,14^. The incidence of GI bleeding in this study was 33.6%, which is consistent with the literature. In clinical practice, GIST patients with GI bleeding should arouse our attention. In our research, GI bleeding has been confirmed as a new independent predictor of RFS and cumulative survival rate for GIST patients, which has been shown in other studies. A study of 596 patients reported that GI bleeding is an independent prognostic predictor for poor RFS (relapse-free survival) in GIST patients^15^. Another study found that GI bleeding is an independent risk factor for GIST recurrence and death and should be considered a significant indicator of poor prognosis^16^. A study from China even suggested using GI bleeding to re-modify the GIST risk stratification system^17^.

Most GIST tumours originate from the bowel wall and grow to both the serosal side and mucosal side. The tumour can restrict the digestive tract mucosa, resulting in altered local mucosal blood supply. Ultimately, barrier damage occurs and causes ulcerative bleeding together with digestive enzyme release. Mucosal ulceration can be seen in 39.6% of GIST patients^12^. Another point of view is that the tumour can invade and erode the mucosal or submucosal blood vessels, cause blood vessel rupture and eventually lead to haemorrhage^18^. Both scenarios may promote poor GIST tumour prognosis.

Fletcher *et al*. proposed the NIH standard in 2002. This standard is based on the two indicators of the maximum diameter of the tumour and the count of the mitotic image, which are used to judge the biological behaviour of GIST and divide the recurrence risk of GIST into four levels^19^. Based on the clinical observation that gastric GIST invasion occurred less frequently than intestinal GIST invasion, Joensuu proposed a modified NIH standard that introduced tumour site and tumour rupture parameters, which has now become a highly practical stratified standard^11^. Currently, the modified NIH standard is the most commonly used diagnostic standard in the clinic. A single-centre 15-year study of 497 GIST patients in 2014 also revealed that NIH risk grade was an independent prognostic factor for both overall survival (OS) (P = 0.026) and RFS (P < 0.001)^20^. According to our research, patients in the very low and low NIH groups exhibited notably higher RFS, which is consistent with previous studies. As shown in Fig. 3B, patients in the intermediate and high NIH groups showed a lower trend in cumulative survival, with no significant differences observed, mainly because of the smaller sample size and shorter follow-up time.

The TT test is a simple and economical test that is widely used in clinical laboratories to ascertain coagulation status. Thrombin leads to the formation of fibrin, which accumulates in cancer tissue and acts as a protective barrier against inflammatory cells^21^. In some conditions, such as DIC, heparin anticoagulant therapy and cancer, the prolongation of TT may decrease the level of fibrinogen or alter its structure and result in overactive fibrinolysis. Fibrinogen layers help tumour cells block natural killer cytotoxicity with thrombin, which can protect tumour cells from the innate immune system^22^. Thus, the clinical consequences of TT reduction can be serious and exacerbate the course of the disease. A study of oesophageal squamous cell carcinoma showed that patients with a lower TT were inclined to suffer hyperfibrinogenaemia and had a poor prognosis in groups stratified by T and N classification as well as metastasis^23^.

Regarding other factor differences, we found that low Hb and high PLR were different between the gastric group and the small intestine group. However, none of them were independent factors. PLR has been demonstrated as a prognostic factor for several solid malignancies, including ovarian^24^, colon^25^ and oesophageal cancer^26^. However, these studies did not provide a reasonable explanation why. Data regarding the association between peripheral blood cells and the prognosis of GISTs are obscure and controversial. A trial in 2016 showed that low Hb and elevated PLR are independent prognostic factors for worse clinical outcome in GIST patients after curative resection^27^.

It is generally known that postoperative hospitalization time is an important indicator to evaluate the quality and short-term outcomes in surgical operations^28–30^. According to our data, patients with small intestinal GIST exhibited higher laparotomy rate than patients in gastric GIST group (*p* < 0.001*), which could lead to prolonged postoperative hospitalization.

Interestingly, our data support the hypothesis that small intestinal GISTs are more aggressive. As shown in Fig. 1, patients with small intestinal GISTs exhibited lower RFS and cumulative survival rates. Additionally, according to the logistic regression analysis, small intestine GIST had a lower TT value, higher GI bleeding rate, advanced NIH category and longer postoperative hospitalization period than gastric GIST. Given the above discussion, these four indicators uniformly indicated a worse outcome and prognosis. In summary, we can conclude that small intestinal GISTs had a higher rate of aggressive features.

We are aware of the limitations of our study. First, this was a single-centre, retrospective study, which could lead to potential selection bias, and the sample size was limited. Second, we focused only on Chinese GIST patients without considering other ethnic groups. Moreover, such short-term follow-up is not long enough because of the relatively better prognosis in GISTs than in other malignant tumours, such as gastric cancer. Considering the advances in surgical technique, instrument and perioperative management strategy during recent years, extension of selection period could lead to major bias between participants. Therefore, we decide to select patients within a shorter period that could improve homogeneity and reduce potential selection bias. Patients with metastatic GIST were not included into our study. The inclusion criteria were patients that received R0 resection, which was not considered as the initial therapy for metastatic GIST.Due to the sample size limitation (only 43 small intestine GIST) of subgroups, subgroup analysis of duodenal jejunal and ileal GIST cannot be performed using statistical methods. Meanwhile, the purpose of this study is to compare the outcome of gastric and small intestine GISTs with initial R0 resection. Patients with preoperative treatment including imatinib, chemotherapy and radiotherapy were not included into this study. Additionally, mutational data were not provided mainly due to the following reasons. 1) The KIT/PDGFRA/BRAF data was missing in patients receiving emergent surgery. 2) Molecular detections of GISTs are expensive and not covered by Chinese Medicare Insurance Policy. Certain patients cannot afford molecular analysis. 3) Only 10 patients (23.3%) in the small intestine group and 12 patients (12.4%) in the gastric group have molecular detections data, which hampers further comparison of molecular features between gastric and intestinal GISTs.

## Conclusions

In conclusion, small intestine GISTs were associated with lower TT, recurrent GI bleeding, advanced NIH category and extended postoperative hospitalization. Nevertheless, future multicentre prospective study are expected to validate our results.
